# Does exposure to asbestos cause prostate cancer? A systematic literature review and meta-analysis: Retraction

**DOI:** 10.1097/MD.0000000000023097

**Published:** 2020-10-30

The article, “Does exposure to asbestos cause prostate cancer? A systematic literature review and meta-analysis”, which published in Volume 98, Issue 3 of *Medicine*, is being retracted because the quality and thoroughness of the literature review, concerns regarding double-counted observations, the inclusion of some cohorts of oil refinery workers, and the potential for bias in the combination of prostate cancer incidence and mortality. Authors were contacted and asked to provide corrections to address the issues, but did not provide such corrections.
